# Exploration of Food-Seeking Behaviour, Food Preparation, and Restrictions to Sufficient Food among the Jahai Sub-Tribe (Indigenous People) in Gerik, Malaysia

**DOI:** 10.3390/ijerph17010348

**Published:** 2020-01-04

**Authors:** Wan Ying Gan, Norhasmah Sulaiman, Leh Shii Law, Nurzalinda Zalbahar, Salma Faeza Ahmad Fuzi, Martin A. Wilkes

**Affiliations:** 1 Department of Nutrition and Dietetics, Faculty of Medicine and Health Sciences, Universiti Putra Malaysia, Serdang 43400, Selangor, Malaysia; norhasmah@upm.edu.my (N.S.); nurzalinda@upm.edu.my (N.Z.); salmafaeza@upm.edu.my (S.F.A.F.); 2 Department of Community Medicine and Public Health, Faculty of Medicine and Health Sciences, Universiti Malaysia Sarawak, Kota Samarahan 94300, Sarawak, Malaysia; lslaw@unimas.my; 3 Centre for Agroecology, Water and Resilience, Coventry University, Ryton Organic Gardens, Wolston Lane, Ryton-On-Dunsmore, Coventry CV8 3LG, UK; martin.wilkes@coventry.ac.uk

**Keywords:** food access, household food security, indigenous peoples, eating behaviours, barriers to food supply, focus group discussion

## Abstract

Access to food has been determined to be a factor that strongly influences the dietary intake and eating habit of indigenous people (*Orang Asli*, OA). This study explored food acquisition and the barriers in obtaining traditional and market foods among the Jahai subtribe (within the Negrito ethnic group) via a qualitative approach. Twenty-eight OA (14 males and 14 females) from Gerik, Perak, Malaysia participated in four focus group discussions (FGDs; two male-only and two female-only groups; seven informants in each FGD). Thematic analysis was adopted to analyse the gathered data. The results found that the Jahai applied both modern (buying and receiving food assistance) and traditional (gathering, farming, fishing, and hunting) methods in obtaining food. The barriers they faced in gathering sufficient food supply included low purchasing power, high demand for food, high transport costs, depletion of food supplies in their surroundings, threats from wild animals on the farm and during food searching activities, and food sharing. Food preparation methods practiced by the OA include roasting and grilling, frying, simmering (*gulai*), and boiling. In conclusion, this study enhances our understanding of the dietary behaviour of the Jahai subtribe and highlights restricted food access among the OA, which demands urgent attention.

## 1. Introduction

As of 2017, the indigenous people in Malaysia (commonly known as *Orang Asli*, or OA, in Peninsular Malaysia) comprised 13.8% of the national population of 31.7 million. A total of 18 ethnic subgroups of OA exist in Malaysia, which are divided into three major tribal groups, namely the Senoi, Negrito (Semang), and Proto Malay (Aboriginal Malay). They account for an estimation of 215,000 or 0.7% of the population of Peninsular Malaysia [1]. The Negrito tribe (3%) are the smallest part of the OA population in Peninsular Malaysia, while the Senoi (55%) are the largest, followed by the Proto Malay (42%) [2]. The Jahai subtribe is one of the six subtribes that fall under the Negrito, who are mainly located in Northeast Perak and West Kelantan. They live along rivers and lakes [3].

Culture, including the types of food and the practices of procurement, preparation, sharing, and eating, is a key determinant of indigenous diets, which has been associated with food insecurity among the OA population [4]. Traditional food can be obtained freely from the surrounding environment without involving any use of money. Traditional foods refer to foods that are harvested, hunted, fished, or farmed by the OA, including various species of plant foods, animals, and animal products, which are believed to have positive impacts on the health status of OA [5]. Studies on aboriginal peoples in Canada have found that traditional foods contain more iron, potassium, and zinc, as well as fewer carbohydrates (especially sucrose), less saturated fat, and less sodium than market foods, which is good for health [6,7].

Despite the small OA population, they appear to have the highest prevalence of obesity (22.8%) when compared to other major ethnic groups in Malaysia, such as the Chinese (7.6%) [8]. In fact, the double-burden of malnutrition was faced by OA households, in which overweight and obese adults (28%) coexisted with underweight (49%) and stunted (64%) children [9]. The OA adults also suffered from hypertension, diabetes mellitus, and dyslipidemia, which are highly linked to the modifiable lifestyle factors, such as dietary intake and physical activity [10]. This poses significantly adverse consequences on their survival, growth, and development, including national economic productivity.

Despite limited evidence on the accessibility and use of traditional foods amongst OA, particularly within the context of Malaysia, these gaps in the literature should be further explored to enhance the aspects of food security and health status among the OA communities. Many local quantitative studies on OA have investigated Senoi and Proto-Malay tribes [9,10,11,12,13,14], while largely omitting the Negrito [15]. As such, the present study aimed to explore food acquisition among the Jahai subtribe, along with the barriers that they have been facing to obtain traditional and market foods in a qualitative manner, in order to better understand food-related activities of the Jahai. This information is crucial for the authorities, as a guide to construct suitable programs for the Jahai to improve their health status and quality of life.

## 2. Literature Review

Having sufficient food is a basic human right. This right does not only mean fulfilling the minimum needs of macronutrients (calorie, protein, and fat) and micronutrients (trace elements and vitamins), but it also involves all the nutritional elements that contribute to active and healthy lifestyle [16]. Food must be available, accessible, and adequate [16]. As a component of food security, food access is defined as ‘*the household’s ability to acquire adequate amounts of food, through one or a combination of own home production and stocks, purchases, barter, gifts, borrowing and food aid*’ [17]. Food access can be achieved through producing one’s own food supply, hunting, fishing, and the gathering of wild foods, as well as purchasing at markets or stores, exchanging through a barter system, or receiving gifts in the form of food [17]. Despite there being sufficient global food production to meet the need of the population [18], an estimated 821.6 million of the world’s population were reported to experience hunger in 2018 worldwide [19]. Both the general population [20] and the indigenous communities [21] are facing issues related to food access, which is linked with the high prevalence of individual and household food insecurity.

The importance of food access has been emphasised due to its influence in dietary patterns. Information from the 2018 Food and Health Survey revealed that American consumers commonly purchased food from supermarkets or super-stores [22]. Additionally, previous studies on the factors influencing food access status of households among the general population were mainly concentrating on financial status [23] and physical environmental factors, such as presence of food outlets nearby residential areas or travel duration to the nearest food outlets [24]. Indigenous people from Malaysia, Australia and Canada appeared to share a similarity in their food purchasing behaviour with the general population, in which they purchased foods from stores such as supermarkets or small grocers [4,15,25]. However, the cost of food was one of the key determinants of food purchases [4]. A local study done by Nurfahilin and Norhasmah [12] found that 28.3% of the OA purchased food on credit and 79.3% of the OA purchased less expensive products or shopped at cheaper stores.

Apart from the cash economy, there are other aspects of food-seeking behaviour among the indigenous people worth exploring, especially those categorised under the traditional food systems. In traditional food system, varying approaches had been practiced by the indigenous people to obtain adequate food, such as gathering, hunting, fishing, and even agricultural activities. Due to the discrepancy in the food-seeking behaviors among the indigenous people, it was expected that the barriers to food access between indigenous people and the general population were also different. For example, a study conducted in Canada revealed that the restriction for aboriginal peoples to carry out traditional food-seeking activities included the abandonment of traditional practices (reduced availability of the traditional foods, unaffordable harvesting tools, loss of culture of hunting activities, and lack of time due to employment) and deterioration of the surrounding environment (contamination of the traditional foods) [26]. As such, coping strategies practiced by indigenous people experiencing household food insecurity have been highly cultural oriented, with an emphasis given to traditional food practices in hunting, fishing, and gathering, as well as food sharing, which are unique and representative of the belief and value of the indigenous people [27]. Food sharing is an important value among indigenous people where they share meals among family members and within communities [4].

A wide range of traditional (e.g., hill paddy, monkey, and mushroom) and modern (e.g., white rice, burger, and watermelon) food items had been identified in the diet of OA in Malaysia [14]. Diet acculturation was observed among them, in which they consumed both traditional food and modern food, yet they still preferred their traditional food [28]. Traditional food continues to be an important component of the diet for indigenous people [26,28]. As environmental and socioeconomic changes continually challenge the indigenous people, the availability of traditional foods is crucial to maintaining their food security and health status [26]. Environmental degradation has been reckoned as a major aspect of the declining consumption of traditional foods amidst the indigenous people, such as erosion of biodiversity, deforestation, hydroelectric dam construction, water pollution, and climate change [29]. The Jahai subtribe has been facing some challenges to access to traditional foods due to dam building and eco-tourism development [30]. Less access to traditional foods leads to a poor diet and poor health in indigenous people [31]. Moreover, the OA in Malaysia, who are originally lived in jungles, have moved out to sub-urban areas [28], due to government policy aiming in improving quality of life of OA by implementing various programs, including resettlement and food aid. Therefore, OA in suburban areas are easily exposed to market foods and different cultures, which may change their food and lifestyle preferences [28]. 

In light of cooking methods, the Aboriginals in Australia were reported to roast food on hot coals, bake in the ashes, and steam in a ground oven via traditional methods. Nonetheless, transformation was observed as new cooking methods, such as boiling and barbecuing, were adopted [32]. Such transformation that substitutes traditional cooking methods with modern ones occurred stemming from colonisation. Amalgamation of traditional and modern ingredients, as well as cooking utensils, was applied in the ground oven technique among Torres Straits Islanders in Australia. Several obvious examples refer to the use of aluminium foil to replace barks and leaves as food parcel wraps and use of plastic cutlery while serving food [33]. During family celebrations, modern cooking methods and foods, namely barbecue, roast, stews, dips, salads, and cakes, had been observed [33]. In Malaysia, the cooking methods of OA have not been widely explored. A study found that 91.8% of Semai OA households in Perak, Malaysia relied on liquefied petroleum gas as the source of cooking fuel, while the other 8.2% used firewood [34]. Meat or flesh food was mainly cooked in bamboo or smoked in the Semai households [34].

In summary, studies on food-seeking behaviour among OA in Malaysia are still in scarcity. Therefore, this study investigated food acquisition and barriers in obtaining traditional and market foods among the Jahai subtribe using the qualitative research approach.

## 3. Materials and Methods

### 3.1. Informants

This qualitative study adopted an illustrative case study approach. A large number of Jahai were reported to reside in Gerik, Perak state, Malaysia, based on the information provided by the Department of Orang Asli Development (JAKOA). A list of names of hardcore poor households (income below MYR 520 per month (≈USD 124)) [35] receiving food aid from JAKOA was obtained from the state JAKOA administrative office in Perak. The village with the highest number of hardcore poor households was identified and purposely selected. There were 60 households with 400 villagers in the selected OA village. The *Tok Batin* (leader of the village) was informed regarding the research activities. Assistance from the *Tok Batin* was acquired to recruit suitable informants, mainly because the *Tok Batin* would have intimate knowledge about the villagers. A total of 28 informants (14 males and 14 females) were selected from the OA village via purposive sampling. Informants with physical and mental disabilities were excluded from this study.

### 3.2. Ethical Clearance and Permission

Prior to data collection, ethical clearance was sought from the Medical Research and Ethics Committee (MREC), National Institutes of Health, Malaysian Ministry of Health (Reference Number: NMRR-17-2922-39130). Permission to conduct this study was obtained from the JAKOA and the Perak State Park Corporation.

A briefing session was carried out to explain the objectives of the research activities to the informants. A written informed consent and permission to audio-record the focus group discussion (FGD) sessions had been obtained from the informants.

### 3.3. Data Collection

The FGDs were conducted in a classroom at a school located in the OA village. Four FGDs (two male-only and two female-only) were conducted to gather information. Each group consisted of seven informants, which met the recommendation by Krueger [36]. The researcher served as the moderator to regulate all processes during the FGD. The language used during the discussion was Malay, the national language of Malaysia. One question was directed to the group at one time. The researcher was also responsible for ensuring that everyone in the group had a chance to express their opinions. The full FGD sessions were audio recorded. Each FGD session took approximately an hour to complete.

### 3.4. Study Instruments

An interview protocol was prepared, and it was divided into two sections: (1) demographic and socioeconomic characteristics, and (2) food-seeking behaviour. Under the demographic section, information pertaining to age, sex, marital status, religion, and household size, was gathered. Meanwhile, the socioeconomic status of the informants, including education level and occupation of informants and their spouses, monthly household income, and monthly household expenditure on food, was obtained.

The FGD was conducted to explore food acquisition based on traditional techniques (scavenging and hunting and gathering) and pastoralism, as well as gardening and modern techniques (purchasing at market). The information on perceived barriers to obtaining food and food preparation methods for the family was also gathered from the FGDs. In order to achieve the objectives of the FGD, several important questions were answered by the informants:

1. Could you please explain how you obtain food for your family?
2. What are the obstacles/difficulties faced to gain food sources for your family?
3. What are the food preparation techniques that you generally use to prepare food for your family?

### 3.5. Data Analysis

A thematic analysis was adopted in this study. The procedure to perform thematic analysis was as prescribed by Braun and Clark [37]. First, verbal data retrieved from the FGDs were transcribed verbatim. Repeated reading was performed by the researcher to familiarise him- or herself with the contents. During the process, several actions, such as searching for meanings and patterns of data, taking important notes, and marking ideas for coding processes, were carried out. Next, coding processes were conducted by using highlighters. Every identified code was matched with the extracted data that demonstrated the characteristic of the code. The next step was to sort the codes into potential themes or sub-themes. Refinement on the themes or sub-themes was needed to decide on the need for combining two themes or separating a theme into two. The extracted data were reviewed to determine their relevance with the themes or sub-themes. A name was given to label the theme in accordance to their characteristics.

## 4. Results

### 4.1. Background of the Informants

The demographic and socioeconomic characteristics of the informants are presented in Table 1. The median age of the informants was 30 years old. Most of the informants were atheist (64.3%), married (82.1%), and had more than four family members (57.1%) in the household. About two in five of the informants (42.9%) and half of their spouses (52.2%) completed secondary education. Approximately half of the informants (50.0%) and their spouses (56.5%) were unemployed. The median monthly household income was MYR 300 (≈USD 72). Almost all the informants (96.3%) had a monthly household income below the poverty line income (<MYR 940). The median income per capita was MYR 50 (≈USD 12), with most of them (92.3%) earning an income per capita below the poverty line income (<MYR 240). Furthermore, the median monthly food expenditure was MYR 100 (≈USD 24).

### 4.2. Focus Group Discussion (FGD)

#### 4.2.1. Question 1: Could You Please Explain How You Obtain Food for Your Family?

Six techniques to obtain foods were reported by the informants. Some techniques were consistently applied by all four groups, such as buying groceries at the market, seeking foods at the forest, and fishing at the nearby lakes and rivers. Foods that were commonly obtained through cash economy were rice, sugar, flour, and condensed milk. Jahai live along the rivers and therefore fish is their main source of food in this study. They obtained foods such as fern shoot and tubers at the nearby forest. Meanwhile, several techniques were only applied among one or two groups of informants, such as hunting animals in the forest, receiving support from non-governmental organisations (NGOs), and planting crops. The themes/sub-themes obtained from the FGD are presented in Table 2.

#### 4.2.2. Question 2: What Are the Obstacles/Difficulties Faced to Gain Food Sources for Your Family?

Barriers to obtain sufficient food included lack of food sources, as well as threats from wild animals on the farm and during food searching activities. These barriers specifically influenced food obtained via traditional food system. Lack of food sources was mainly due to the deterioration of the surrounding environments, which threatened the reproduction and survival of traditional food species. Besides, centralised resettlement had exhausted the sustainability of the ecological system at the nearby environments due to high competition and demand from the other resettlements. Depleted food sources had forced the Jahai to move further from the resettlement and deeper into the forests, so as to fulfil their quest in finding sufficient food. 

Some groups added that low purchasing power and high transportation costs as barriers to secure adequate food supply for the households. These barriers reflect the hardships borne by the Jahai from the stance of cash economy. Most of the Jahai were involved in small-scale self-sufficient agricultural activities that did not promise lucrative return. They claimed that sometimes they purchased food on credit at the shops in Gerik town when they had not enough money. Besides, there was no any food outlet in this OA village and the villagers had to travel by boat for two hours to the nearest Gerik town to buy the necessity. They need to spend high cost for fuel due to the long distance between their village and the nearby town, thus leading to high transportation cost.

Sharing food with others and high demand for food were the other barriers observed in securing adequate food for the family in this study. It has been a tradition amongst the OA to establish strong relationship with their family members and neighbours through food sharing. Whenever a household at the village had the ability to purchase food, they would share the food items with other villagers with insufficient food. Since rice has been the staple food for OA, it was commonly purchased at Gerik town. A 10 kg pack of rice might last only for a few days due to high demand from the big number of household members. It is worthy to highlight that many OA households had extended family living together in a house. In this study, 57.1% of the households had more than four family members (Table 1). Meal skipping was a coping strategy when they faced insufficient food supply. Low purchasing power and high demand for food worsened the food condition of the Jahai. Table 3 presents the barriers identified in this study. 

#### 4.2.3. Question 3: What Are the Food Preparation Techniques That You Generally Use to Prepare Food for Your Family?

Four food preparation methods were usually practiced by the informants. Examples of cooking methods were roasting and grilling, frying, simmering (*gulai*) (gravy mixed with spices), and boiling. The simmering method is different from the boiling method; simmering is a method to cook food gently and slowly in liquid at a temperature just below the boiling point. The product of simmering, the *gulai*, appeared to be thicker than soup and less watery, due to the use of spices and coconut milk. Cooking oil and water were the main medium for cooking among OA. Details regarding the food preparation methods by OA are presented in Table 4.

## 5. Discussion

The study outcome reveals the food-related activities of the Jahai subtribe, so as to enhance our understanding of the uniqueness of the Jahai subtribe in their food activities, despite sharing certain similarities with the behaviours of the general population. Six methods are applied by the Jahai subtribe to obtain food, including buying, searching for edible plants in the jungle, fishing at nearby lakes and rivers, hunting wild animals, farming, and receiving food assistance from the government or NGOs. Food-seeking behaviours displayed by OA could be divided into two categories, namely modern (buying and receiving food assistance) and traditional practices (food gathering, farming, fishing, and hunting). The Jahai were not able to escape from globalisation, and have undergone the processes of assimilation and modernisation [39]. Similar findings have been reported by prior studies, in which both market (e.g., ready-to-eat cereals, yellow noodles, and broccoli) and traditional (e.g., frog, *petai*, and *pucuk paku*) food items were found in the diet of OA [14,15]. However, such behaviour must be monitored, as diet acculturation might exert a negative impact on the health of OA, such as overweight and obesity, elevated waist circumference, and increased body fat percentage [28]. Food supply was also obtained by gaining food assistance from government and NGOs. Apart from Malaysia, food assistance is also provided to OA who suffer from food insecurity in Canada [40]. It is integral to acknowledge that the provision of food assistance is not a long-term solution to overcome household food insecurity among OA.

The ability to obtain food through modern and traditional practices has not shielded the OA from household food insecurity. The study findings offer evidence on the barriers to obtaining sufficient food. Similar conditions were reported in other local quantitative studies [11,12]. For instance, the high prevalence of household food insecurity was reported, in which 82.9% of the Mah Meri (within the Senoi ethnic group) households in Kuala Langat, Selangor, experienced food insecurity, with 29.3% as household food insecurity, 23.4% as individual food insecurity, and 30.2% as child hunger [11]. Another study among OA in Gombak, Selangor, reported that 88.0% of the households experienced food insecurity, whereby 48.9% was attributed to household food insecurity, 21.7% as individual food insecurity, and 17.4% for child hunger [12]. The findings indicate that household food insecurity among OA should be given attention, due to its high prevalence.

Both modern and traditional practices have their own restrictions in food-seeking activities. Under modern practices, this study found that low purchasing power was a constraint to purchasing sufficient food. Low purchasing power was attributed to low socioeconomic status, such as high involvement in self-employed occupations and low education qualifications among the informants in this study. The contribution of low socioeconomic status on household food insecurity was common, as reported in the past studies [11,12]. Inadequate income leads to low purchasing power, which not only limits food supply, but also the variety of food to fulfil the need of the household members [11]. High demand of the household members, especially among those from a large-size household, worsens the situation, as those in larger households are expected to receive less and lower-quality food when compared to those with a smaller household size [41]. This present study found that some informants were required to travel to a nearby town to purchase food once a month. They claimed that they spent two hours to travel by boat to the nearby town. Along with limited income, high transportation costs increased the burden on the financial status of OA and further depleted their purchasing power [42].

Traditional food-seeking practices of OA were once believed to be a solution to household food insecurity, as their traditional knowledge guided them to search for sufficient food to meet the demands of their household members [43]. However, the destruction imposed on their surrounding environments, due to deforestation and the pollution of water supplies, depleted access to fish in the rivers/lakes, wild edible plants, and animals in the jungle [15,29]. Changes in lifestyle have also motivated a large fraction of OA to abandon their traditional food seeking activities. This has led to a loss of hunting and gathering knowledge from one generation to another. Such a group would become involved in cash economic activities in the nearby town. A similar phenomenon was reported in the Colombian Amazon [44] and Canada [26], due to modernisation and urbanisation.

Another challenge to traditional food-seeking practices has been threats from wild animals on both plantation and food-seeking activities in the OA’s surrounding environment. Low output from agricultural activities and hunting exerted significant pressure on the OA, who relied solely on traditional practices to support the food system of their household members. Such information is deemed to be accurate, as the findings are consistent with those reported by Law et al. [15]. OA are willing to share food with each other during difficult times, as everyone in the village has collective ownership of the available food, be it edible plants, fish, or wild animals [43]. The practice of food sharing was also reported among the aborigines in Canada and Australia [40]. Turning to the present study, under normal circumstances, food sharing can help to relieve the burden of food insecure households; however, under abnormal circumstances of food insecurity, sharing of food would burden both households.

As an independent cultural entity, the OA have several distinctive cuisines in terms of the ingredients used. However, similarities have been observed in food preparation methods between the OA and the general population. Understanding the frequent use of certain food preparation methods is essential, as food preparation methods have been found to influence the nutritional quality of food [44]. The impact of food preparation methods on the nutritional status of the Jahai is indeed an interesting topic to explore in future studies.

This study has several limitations. First, only one ethnic group, namely the Negrito, was recruited to be the participants in the present study. Future studies could involve the other two ethnic groups of the OA (Senoi and Proto Malay) from various other locations, so that a comparison can be made to identify both the variances and similarities in their food-related activities. Second, only one technique, which is the FGD, has been employed in this study. The accuracy of the information provided by the informants could not be determined. Thus, apart from FGDs, other research methods, such as observation or in-depth interview, should be incorporated in future studies.

## 6. Conclusions

The attainment of information regarding food-seeking behaviour and food preparation methods enhances our understanding of the dietary behaviour of the Jahai subtribe, which has not been widely explored previously. The food habits of the OA seem to differ from those of the general population, which depends solely on the market to obtain food. The findings of this study are practically and clinically relevant, especially in Malaysia, where there are different subtribes of OA with varied food-seeking behaviours that indirectly affect their food preparation and selection, and ultimately their health status, food security, and overall quality of life. Government and NGOs should collaborate to increase the accessibility of traditional food as a means of achieving food security for the OA. The findings in this study may serve as a guideline to policy makers and healthcare practitioners who are responsible in planning viable and strategic intervention for the OA, so as to improve the dietary status of the OA. It is crucial to take into account the context of food access for the OA, which is distinctive with the one of the general populations in the planning process of intervention programs, primarily to enhance its effectiveness. Several issues related to environmental degradation, the costs and benefits of food sharing, and details on food purchases among the OA warrant further exploration.

## Figures and Tables

**Table 1:** Background of the informants (*n* = 28).

Variables	Median (IQR)	*n*	%
Age (years)	30.00 (13.25)		
17–30		14	50
31–44		11	39.3
>45		3	10.7
Sex			
Male		14	50
Female		14	50
Religion			
Islam		10	35.7
Atheist		18	64.3
Marital status			
Single		2	7.2
Married		23	82.1
Widow		3	10.7
Informant’s education level			
No formal education		8	28.6
Primary education		8	28.6
Secondary education		12	42.8
Spouse’s education level (*n* = 23)			
No formal education		4	17.4
Primary education		7	30.4
Secondary education		12	52.2
Household size ^a^	6 (4)		
≤4		12	42.9
>4		16	57.1
Informant’s occupation			
Housewife/unemployed		14	50
Fisherman		11	39.3
Forest product seeker		3	10.7
Spouse’s occupation status (*n* = 23)			
Housewife/unemployed		13	56.5
Fisherman		4	17.4
Fisherman and forest product seeker		2	8.7
Forest product seeker		4	17.4
Monthly household income (MYR) (*n* = 27) ^b^	300.00 (350.00)		
<940		26	96.3
≥940		1	3.7
Income per capita (MYR) (*n* = 27) ^b^	50.00 (87.50)		
<240		25	92.6
≥240		2	7.4
Food expenditure per month (MYR)	100.00 (150.00)		

^a^: Average household size was 4, based on the average family size (persons) from the Population and Housing Census of Malaysia 2010 [38]; ^b^: income category based on the Poverty Line Income (PLI) in Peninsular Malaysia by the Economic Planning Unit [35]. IQR: interquartile range; MYR: Ringgit Malaysia; USD 1 = MYR 4.19 as of 1 October 2019.

**Table 2:** Sub-themes of techniques used to obtain foods and feedback from informants.

Sub-Theme	Feedback from Informants
Buying from local grocery shops or food outlets	“Buy groceries such as sugar, rice, condense milk, raw chicken, raw fishes, cabbage, mustard green and bean sprouts at Banding Island and Gerik town”
	“Buy groceries such as rice, sugar, flour and milk at Banding Island and Gerik town once a month”
	“Buy groceries such as rice, milk, sugar, onion and bread at Banding Island and Gerik town”
	“Buy rice, sugar, condense milk, cooking oil, salt, cabbage, mustard green, raw sea fishes and legumes one a month at Gerik town”.
Seeking edible plants in the jungle	“Seeking *ubi garam* and *sayur bayas*, *sayur cukur*, fern shoot, and forest fern shoot at Royal Blum Forest once a week”
	“*Ubi garam* (boiled or roasted before eating), *umbut bayas* and *cucur* (vegetables) from nearby forest”
	“Seeking tubers such as *ubi garam*, *tuleng*, and *daran*, as well as vegetables such as *umbut payas*, *keliwan*, *lahau*, fern shoot and *hendi* at nearby forest”
	“Examples of forest products such as *ubi garam*, *ubi pasir*, *ubi tali*, *umbut bayas*, *gaduk*, *bayan gadai*, and *saiyen*”
Fishing at nearby lakes and rivers	“Fishing at Terjun River and Ampang River [1 km from the village]. The fish mostly obtained is *ikan Tengas*”
	“Fishing at Blum River and Ampang River”
	“Fishing at nearby river every day. Manage to obtain 10 to 15 fishes such as *ikan patoi*, *ikan kaloi*, *ikan tembiras* and *ikan baung*”
	“Going out for fishing every day by using fishing nets. Examples of fishes obtained from fishing are *ikan tengik, ikan terbul, ikan patung*, *ikan mayang*, *ikan kaloi*, *ikan selak* and *ikan hujan*”
Hunting for animals in the forest	“Very rarely (once in a month) we hunt at the forest unless desperately need to do so. Examples of animals obtained from the forest are chicken and mousedeer”
	“Goes out to hunt every day from morning until late evening by using a blowgun. Examples of foods obtained from the forest are eagles, birds and mousedeer”
Receive aids from organisation/bodies	“Receive aids from NGO”
	“Receive groceries support from JAKOA once in two months”
Farming	“Examples of crops are tubers, banana and sugar cane”

**Table 3:** Sub-themes of barriers in obtaining foods and feedback from informants.

Sub-Theme	Feedback from Informants
Lack of food sources	“Not much food in the nearby river”
	“The forest products were depleted”
	“Forest products were getting limited”
Threats from wild animals	“The crops were threatened by wild animals such as wild pigs and elephants”
	“Wild animals destroyed the crops”
	“Cannot plant vegetables because of the presence of wild animals”
	“Wild pigs ruined the crops”
Low purchasing power	“Buying food on credit at shops in Gerik town”
High transportation costs	“Increment in fuel leads to high transportation cost. We spend two hours to travel by boat to go to Gerik town. Usually, the food storage would run out within five to six times per month, and the neighbours around would help and share their foods”
Food sharing with others	“Sharing foods with others is a culture here. We share the foods with neighbours if we are able to go out and buy foods”
High demand for food	“If the food supply runs out, the frequency of meals intake daily will decrease. We only eat once daily”
	“Rice runs out almost every week, so we always go out fishing”

**Table 4:** Cooking method sub-theme and feedback from informants.

Sub-Theme	Feedback from Informants
Roast/grill	“Usually roast or grill the fish”
	“Preferred roast/grill without cooking oil”
	“Roast or grill the foods if cooking oil was not available”
Simmer (*gulai*)	“Heat the mixture of cooking oil, water and salt”
	“Added a bit of curry powder in the mixture of cooking oil, water and salt”
Fry	“Onion was sautéed in hot cooking oil”
	“Fried the foods by using cooking oil”
	“Add water into the stir-fried vegetables”
Boil	“Always cook foods by using the boiling method”
	“Cook soup dishes, in which water and salt are the main ingredients”
